# Potential benefits of applying the GebStart-tool for advising primiparous women during early labor: A comparative study

**DOI:** 10.18332/ejm/223604

**Published:** 2026-07-03

**Authors:** Susanne Grylka-Baeschlin, Nadine Pauli, Catherine Rapp, Carola Baumgarnter, Clizia Iseppi, Nele Struebing, Linda Karg, Gabriela Minati, Leonhard Schaeffer, Olav Lapaire, Markus Hodel, Gabriella Stocker, Nina Kimmich, Leila Sultan-Beyer, Antonia Nathalie Mueller

**Affiliations:** 1 Research Institute of Midwifery and Reproductive Health, School of Health Sciences, ZHAW Zurich University of Applied Sciences, Winterthur, Switzerland; 2 Midwifery Division, School of Health Professions, Bern University of Applied Sciences, Bern, Switzerland; 3 Division of Obstetrics and Prenatal Diagnostics, Cantonal Hospital of Baden, Baden, Switzerland; 4 Women’s Clinic, University Hospital of Basel, Basel, Switzerland; 5 Women’s Hospital, Cantonal Hospital of Lucerne, Lucerne, Switzerland; 6 Gynaecology and Maternite, Stadtspital Zurich Triemli, Zurich, Switzerland; 7 Department of Obstetrics, University Hospital Zurich, Zurich, Switzerland; 8 Division of Obstetrics and Prenatal Diagnostics, Cantonal Hospital, Winterthur, Switzerland; 9 Medical Department, University of Zurich, Zurich, Switzerland

**Keywords:** latent phase of labor, birth, obstetric outcomes, caesarean section instrument development

## Abstract

**INTRODUCTION:**

Early labor care is complex because of women’s individual needs. To consider varying experiences and distinguish between parturients who are well at home and those who require increased support, the GebStart-tool was developed. It intends to advise primiparous women during early labor, enhance the quality of care, and improve perinatal outcomes. The aim of this study was to assess the potential benefits and risks of applying the GebStart-tool.

**METHODS:**

Applying the preliminary version of the GebStart-tool, we compared labor and birth data from n=303 study participants with spontaneous onset of labor with baseline data of n=1635 births that occurred in the six months preceding the study across six centers. Descriptive statistics and odds ratios were calculated.

**RESULTS:**

GebStart-study participants had significantly lower odds for labor augmentation with oxytocin (OR=0.65; 95% CI: 0.51–0.84, p<0.001), epidural analgesia (OR=0.56; 95% CI: 0.43–0.72, p<0.001), and cesarean section (OR=0.52; 95% CI: 0.36–0.76, p<0.001) compared to baseline data. In contrast, the odds for opioid administration (OR=1.37; 95% CI: 1.02–1.83, p=0.028) and a spontaneous vaginal birth (OR=1.33; 95% CI: 1.02–1.72, p=0.028) were significantly higher. Apgar scores at one minute and arterial umbilical cord pH did not differ substantially between groups.

**CONCLUSIONS:**

Compared to baseline data, GebStart-study participants had a higher chance of fewer intrapartal interventions and a spontaneous vaginal birth. Therefore, using the GebStart-tool seems promising for improving labor and birth outcomes. In a future larger study, the effectiveness of applying the final version of the GebStart-tool should be investigated.

**CLINICAL TRIAL REGISTRATION:** The study was registered in the Swiss National Clinical Trials Portal and the German Clinical Trial Register.

**IDENTIFIERS:** SNCTP000004555 and DRKS00025572

## INTRODUCTION

Individualized early labor care may provide a foundation for a favorable labor and birth process as well as positive maternal and neonatal outcomes. Early labor is the first phase after the onset of labor, which, together with the active phase of labor, constitutes the first stage of labor^1^. It is characterized by a variety of symptoms^2^. Its management is often not well-experienced by parturients^3-6^ and is also challenging for healthcare professionals^7,8^. Pregnant women at the onset of labor, especially those expecting their first child and experiencing a prolonged early labor, frequently seek professional support before active labor begins^5,8^. Concurrently, research findings show more frequent obstetric interventions and poorer obstetric outcomes if women are admitted to the hospital early during the labor process^9-11^. For this reason, midwives and other health professionals act as gatekeepers to delay hospital admission, which can lead to anxiety and fear in insecure parturients^12,13^. While home is the best place for many women, some feel unsafe and need reassurance or pain management^8,14^.

Several studies support measures such as telephone calls, home visits, or algorithms, which were implemented and tested to delay hospital admission and improve labor care^15-17^. In a Cochrane review including five trials with 10421 pregnant women and a cluster randomized trial with 2183 women, Kobayashi et al.^17^ investigated the effect of assessment and support interventions during early labor. The investigated trials were conducted in the UK, Canada, and the USA, and did not provide clear evidence for the reduction of cesarean section and instrumental birth rates. However, women in the early assessment group received epidural and oxytocin augmentation slightly less often and reported higher satisfaction with their care^17^. The authors of that review concluded that the interventions did not sufficiently address women’s individual needs, thereby limiting their effectiveness and indicating that these approaches have not yet yielded adequate solutions.

In recent years, there has been a growing awareness of the need for new, more women-centered approaches to early labor care^3,14,17^. Measures such as support via video calls, apps and specific assessment instruments were developed^18-21^. Among other offers, the GebStart-tool, a standardized questionnaire was designed to advise primiparous women during early labor about their best place to stay^20,22^.

The application of this tool aims to identify parturients with increased support needs, while reassuring others to remain at home. In contrast to previous instruments, such as the algorithm developed by Cheyne et al.^16^, the GebStart-tool considers not only contraction patterns and vaginal discharge but also additional factors, including wellbeing at home, sleep, nutrition, and the availability of support. This approach enables a more individualized and woman-entered assessment of the parturient’s needs. Furthermore, the tool is also suitable for telephone consultations, as the assessment does not require physical examination. The final GebStart-tool with 15 items was designed and preliminarily validated^20^; however, the effectiveness of the GebStart-tool was not yet reported.

The aims of this part of the study were therefore to investigate: 1) potential benefits and risks of the application of the GebStart-tool in primiparous women during early labor to improve labor and birth outcomes; and 2) differences between study sites to assess robustness of results across institutions.

## METHODS

### Study design and setting

Within the GebStart-study, where we developed and preliminarily validated a tool for advising primiparous women about their best place to stay during early labor^20,22^, we conducted a comparative observational study.

Data from participants in the main phase of the GebStart-study (1 May 2022 to 31 August 2023) in six study centers in the German part of Switzerland^20^ were compared with baseline data collected at the same six study sites during the six months preceding recruitment for the pilot phase GebStart-study (1 September 2021 to 28 February 2022). The multicenter study was conducted in six study sites to enable the target sample size for the GebStart-study to be reached within a year. Additionally, this provided the opportunity to use the instrument in different obstetric teams and assess the robustness of results across study centers. The project was registered in the Swiss National Clinical Trials Portal (SNCTP000004555, 27 July 2021) and the German Clinical Trial Register (DRKS00025572, 28 July 2021). For reporting the results of this observational study, the Strengthening the Reporting of Observational studies in Epidemiology (STROBE) guidelines were followed (Supplementary file)^23^.

### Ethics

Ethical approval was obtained from the Ethics Committees of Zurich as well as North-western and Central Switzerland (Approval number BASEC-Nr. 2021-00687; Date: July 2021). Eligible women for study participation were informed verbally and in writing about the purpose of the study and their right to withdraw at any time. They gave written consent to participate in the study. The baseline data were transmitted in an aggregated, anonymized form to the study office.

### Development of the GebStart-tool

For the development of the GebStart-tool, a pool of 99 initial items was generated based on findings from four focus group discussions^5,24^ and a scoping review with an extensive literature search^2,8^. The content and face validity of the initial items were assessed through qualitative and quantitative evaluation by an international multidisciplinary expert panel. In the quantitative part, relevance and clarity of items were assessed by n=8 experts, and items were reduced to 32. The preliminary GebStart-tool was subsequently applied in six hospitals with n=394 primiparous women^20,22^. Due to the formative and complex nature of the GebStart-tool, factor analysis was considered inappropriate, and alternative statistical analyses were applied. The reduction to 15 items and the preliminary validation were conducted using response distribution analyses, adjusted Cox regression models with care needs over time as outcomes, and adjusted multinomial regression models predicting care decisions^20^. The comprehensive development process of the GebStart-tool has been outlined in detail elsewhere^20,22^.

### Study population

Inclusion criteria for the GebStart-study were: primiparous women aged ≥18 years with a singleton fetus in cephalic presentation, who had not scheduled an elective cesarean section or planned induction of labor, who spoke sufficient German, and who gave birth in one of the six GEBSTART study centers^22^. The study focused on primiparous women due to their higher rates of early hospital admission and adverse outcomes compared to multiparous women^25^.

Among the n=627 women recruited during their pregnancy at six study sites during the main phase of the GebStart-study, the tool specifically developed for this study was applied to n=394^20^. High dropout rates were anticipated during the planning phase of the project due to unforeseeable cesarean sections and labor inductions at the time of recruitment^22^. However, loss of follow-up was higher than expected (37% vs 25%), also because the tool was not completed in n=95 women (15.2%) due to urgent admission, high workload in the labor ward, or because completing the tool was forgotten^20^. Additionally, some of the Gebstart-participants (n=91) had symptoms of early labor at home, called the hospital midwives who completed the GebStart-tool, but subsequently labor was induced, or a cesarean section was performed before active labor started. These GebStart-study participants would not have been identified as parturients with spontaneous onset of labor with the selection criteria used for the baseline data. Therefore, for reasons of comparability, these women were excluded from these analyses, and n=303 women with spontaneous onset of labor formed the group of interest in this comparative study.

Baseline data for comparison were collected in all six study sites and comprised all primiparas aged >18 years with a spontaneous onset of labor from 37+0 weeks of pregnancy who gave birth within a six-month period prior to recruitment in the study centers of the GebStart-study. Across all six study sites, data from n=1635 births were included in the comparison group.

The required sample size for the GebStart-study was calculated based on the needs for scale development. However, the sample was also large enough to allow for comparisons between interventions and events with a prevalence of >5%^26^.

### Measures and variables

Sociodemographic and perinatal data of Gebstart-participants were collected in a REDCap^®^ database, which included a case report form for sociodemographic and labor and birth data as well as self-reported antenatal and postnatal online questionnaires as^20,22^. Data used for these comparative analyses were exported from the case report form, which was completed by the study midwives in the study centers based on the electronic medical record. Questions in the case report form were all set to mandatory to ensure completeness of data.

Baseline data were collected by the same study midwives at the participating centers and was also extracted from medical records. This was accomplished either through the documentation system’s electronic data extraction functionality or by manually reviewing the hospital’s birth register to identify parturients who met the inclusion criteria and had given birth within the six months preceding the recruitment phase of the GebStart-study. Data were then manually extracted from the electronic medical records and entered into an Excel sheet to calculate the mean age and frequency of interventions and events. Variables of interest were the number of women who gave birth during the six-month period before recruitment of the GebStart-study and who met the inclusion criteria of the study, as well as median age and number of women with gravidity >1, as sociodemographic and obstetric history-related characteristics to compare study groups. Additionally, the study focused on the following labor and birth outcomes: synthetic oxytocin administration, amniotomy, opioid administration, epidural analgesia, spontaneous vaginal birth, instrumental vaginal birth, cesarean section after onset of active labor, Apgar score <7 after one minute, and umbilical artery pH <7.15. Gravidity was included because previous pregnancies potentially impact cervical maturation and the selected outcomes were factors described in the literature that are affected by early hospital admission^9-11^. These data were transmitted to the study office in an anonymized, aggregated form to meet ethical requirements.

### Statistical analysis

Descriptive analyses were calculated using relative frequencies for categorical variables and means, medians, and ranges for metric variables. Baseline data were transmitted in aggregated form. The variable age was continuous in the study and in the baseline date. However, for reasons of anonymity in the baseline data, only the mean was transmitted. As the continuous variable age in study participants showed no skewness in the histogram and the mean and median were comparable, it was considered appropriate to compare means between groups. Labor and birth outcomes were collected as dichotomous variables in both datasets. Absolute and relative frequencies were available or could be computed with the group sizes. Odds ratios, 95% confidence intervals, and p-values of events were calculated using contingency tables with the absolute frequencies of exposed (GebStart-tool applied) and not exposed cases, as well as the number of events and no events, and applying the Stata command cci (conservative confidence interval). Data for study participants did not show any missing values, since the variables were set to mandatory in REDCap^®^. For two outcomes (gravidity >1 and opioid administration), data were missing from one site each in the baseline data. These characteristics were therefore compared only across the five study sites that provided the relevant information. Because of the aggregated baseline data, no multivariable analysis to adjust the odds ratios could be calculated.

Statistical analyses were conducted using Stata version 17 (StataCorp, College Town, USA), with statistical significance determined at a threshold of p<0.05.

## RESULTS

A total of n=303 GebStart-study participants were included in these analyses and compared with n=1635 women, who would have been eligible for the study and had given birth within the six months prior to the initiation of data collection for the study.

### Sociodemographic characteristics

GebStart-study participants were, on average, 1.2 years older than parturients in the baseline data (32.9 vs 31.7 years) (Table 1). Due to the aggregated baseline data, no standard deviation could be calculated for this variable, and the significance of the age difference could not be assessed. Due to the study’s inclusion criteria, all women in both groups were primiparae. However, there were significantly more primigravidae in the study participants group compared to baseline data (84.2% vs 77.6%, p=0.011). No further sociodemographic data were collected in the baseline data.

**Table 1:** Sociodemographic characteristics of participants of the X-study compared to baseline data, comparative observational study, Switzerland, 2021–2023 (N=1938)

*Characteristics*	*Participants with tool applied* *n (%)*	*Baseline data all study sites* *n (%)*	*Difference*
**Total**, n	303	1635	
**Age** (years), mean (range), median	32.9 (20.0–41.0) 33.0	31.7	1.2 years
**Nationality Swiss**	213 (73.5)		
**Parity primiparae**	303 (100)	1635 (100)	0 %
**Gravidity primigravidae^a^**	255 (84.2)	1099 (77.6)	6.6 %*

a Data regarding gravidity >1 was missing in the baseline data of one site.

* p<0.05.

### Labor and birth-related characteristics

Participants of the GebStart-study had more often an amniotomy than parturients in the baseline data (17.6% vs 14.2%, p=0.136), but the difference was not significant (Table 2). Oxytocin administration for labor augmentation was significantly less frequent in study participants compared to baseline data (48.7% vs 59.1%, p<0.001). While the proportion of GebStart-participants receiving opioids was significantly higher compared to baseline data (28.3% vs 22.1%, p=0.028), epidural analgesia was significantly less often administered (44.7% vs 59.1%, p<0.001). Additionally, the rate of vaginal birth was higher among study participants, but the difference was only significant for spontaneous vaginal birth (spontaneous vaginal birth: 62.7% vs 55.9%, p=0.028; and instrumental vaginal birth: 24.8% vs 22.3%, p=0.342). Consequently, cesarean section was significantly less frequent among participants in the GebStart-study than in baseline data (12.5% vs 21.5%, p<0.001). Lower Apgar scores after one minute and arterial umbilical cord pH were more frequent in study participants than in baseline data, but the differences were not significant (Apgar 1 <7: 12.9% vs 11.0%, p=0.347 and arterial umbilical cord pH <7.15: 14.9% vs 12.2%, p=0.638).

**Table 2:** Labor and birth related characteristics of participants of the X-study compared to baseline data, comparative observational study, Switzerland, 2021–2023 (N=1938)

*Characteristics*	*Participants with tool* *applied* *n (%)*	*Baseline data all study* *sites* *n (%)*	*Difference (%)*
**Total**, n	303	1635	
**Gestational weeks at birth**, median (range)	40.0 (35.9–41.7)		
**Amniotomy**	53 (17.6)	232 (14.2)	3.4
**Labor augmentation with oxytocin during labor**	147 (48.7)	966 (59.1)	-10.4***
**Opioid administration**	85 (28.1)	282 (22.1)^a^	6.0*
**Epidural analgesia**	135 (44.7)	967 (59.1)	-14.4***
**Mode of birth**			
Spontaneous vaginal	190 (62.7)	914 (55.9)	6.8*
Instrumental vaginal (ventouse)	75 (24.8)	364 (22.3)	2.5
Cesarean section during labor	38 (12.5)	351 (21.5)	-9.0***
**Birth weight** (g), median (range)	3315 (2340–4440)		
**Apgar 1 <7**	39 (12.9)	180 (11.0)	1.9
**Arterial umbilical cord pH <7.15**	40 (14.9)	200 (12.2)	2.7

a Opioid administration is missing in the baseline data of one site.

* p<0.05,

**p<0.01,

*** p<0.001.

### Odds for intrapartal interventions and birth outcomes

Study participants had a non-significantly increased likelihood of an amniotomy being performed during labor compared with baseline data (OR=1.28; 95% CI: 0.91–1.79) (Table 3). The odds for labor augmentation with oxytocin were significantly lower than in baseline data (OR=0.65; 95% CI: 0.51–0.84). For pain management, study participants were 1.37-fold more likely to receive opioids (OR=1.37; 95% CI: 1.02–1.83), but the odds for an epidural analgesia was 45% lower than in baseline data (OR=0.56; 95% CI: 0.43–0.72). Whereas study participants had a 1.33-fold increased chance of a spontaneous vaginal birth (OR=1.33; 95% CI: 1.02–1.72), the odds of a cesarean section during labor were 48% lower than in baseline data (OR=0.52; 95% CI: 0.36–0.76). The likelihood of a lower Apgar score at one minute and of an arterial umbilical cord pH was higher among study participants, but the results were not significant.

**Table 3:** Odds ratios of intrapartal interventions and birth outcomes in study participants compared to baseline data, comparative observational study, Switzerland, 2021–2023 (N=1938)

*Variables*	*OR*	*95% CI*	*p*
**Amniotomy**	1.28	0.91–1.79	0.136
**Labor augmentation with oxytocin during labor**	0.65	0.51–0.84	<0.001
**Opioid administration**	1.37	1.02–1.83	0.028
**Epidural analgesia**	0.56	0.43–0.72	<0.001
**Mode of birth**			
Spontaneous vaginal	1.33	1.02–1.72	0.028
Instrumental vaginal (ventouse)	1.15	0.85–1.54	0.342
Cesarean section during labor	0.52	0.36–0.76	<0.001
**Apgar 1 <7**	1.19	0.80–1,74	0.347
**Arterial umbilical cord pH <7.15**	1.09	0.74–1.58	0.638

### Differences between study sites

Results of the six GebStart-study centers are shown in Table 4. Even though the numbers across the sites were too small for comparative testing, some results were quite robust. In all sites, the age of study participants was higher than in the baseline data. The odds for performing an amniotomy were higher in study participants compared to baseline data in four sites. Furthermore, oxytocin administration for labor augmentation was lower in study participants than in the corresponding baseline date in five centers, and the use of epidural analgesia was lower in five sites. The comparison of birth modes was stable too: spontaneous vaginal birth of study participants was higher in five, and cesarean section was lower in all six study centers compared to baseline data. The differences in neonatal outcomes, in contrast, were more heterogeneous.

**Table 4:** Comparison of sites of the X-study between study participants and baseline data, comparative observational study, Switzerland, 2021–2023 (N=1938)

*Characteristics*	*Site 1*		*Site 2*		*Site 3*		*Site 4*		*Site 5*		*Site 6*	
	*Study* *data* *n (%)*	*Baseline* *data* *n (%)*	*Study* *data* *n (%)*	*Baseline* *data* *n (%)*	*Study* *data* *n (%)*	*Baseline* *data* *n (%)*	*Study* *data* *n (%)*	*Baseline* *data* *n (%)*	*Study* *data* *n (%)*	*Baseline* *data* *n (%)*	*Study* *data* *n (%)*	*Baseline* *data* *n (%)*
**Total**, n	61	208	54	218	24	211	83	400	41	237	41	361
**Age** (years), mean	32.5	30.7	32.4	31.8	34.3	31.9	33.3	32.1	32.4	31.1	33.0	32.2
**Gravidity primigravidae**	53 (86.9)	174 (83.7)	45 (83.3)		18 (75.0)	167 (79.1)	72 (86.8)	315 (78.8)	35 (85.4)	180 (75.9)	32 (80.0)	263 (72.9)
**Amniotomy**	7 (11.5)	34 (16.3)	10 (18.9)	38 (17.4)	4 (16.7)	27 (12.8)	16 (19.3)	33 (8.3)	10 (25.0)	32 (13.5)	6 (15.0)	68 (18.8)
**Labor augmentation with oxytocin during labor**	32 (52.5)	101 (48.6)	17 (31.5)	144 (66.1)	12 (50.0)	125 (59.2)	37 (44.6)	196 (49.0)	19 (47.5)	160 (67.5)	30 (75.0)	240 (66.4)
**Opioid administration**	26 (42.6)	83 (39.9)	19 (35.2)	42 (19.3)	5 (20.8)	42 (19.9)	10 (12.1)	48 (12.0)	7 (17.1)	67 (28.3)	19 (46.3)	
**Epidural analgesia**	30 (49.2)	103 (49.5)	22 (40.7)	136 (62.4)	11 (45.8)	142 (67.3)	25 (30.1)	187 (46.8)	19 (47.5)	137 (57.8)	28 (70.0)	262 (72.6)
**Mode of birth**												
Spontaneous	38 (62.3)	133 (63.9)	29 (53.7)	110 (50.5)	16 (66.7)	113 (53.6)	60 (72.3)	242 (60.5)	29 (70.7)	154 (65.0)	18 (45.0)	162 (44.9)
Instrumental*	15 (24.6)	39 (18.8)	22 (40.7)	65 (29.9)	5 (20.8)	50 (23.7)	11 (13.3)	56 (14.0)	7 (17.1)	40 (16.9)	15 (37.5)	114 (31.6)
Cesarean section	8 (13.1)	36 (17.3)	3 (5.6)	43 (19.7)	3 (12.5)	48 (22.7)	12 (14.5)	102 (25.5)	5 (12.2)	43 (18.1)	7 (17.5)	79 (21.9)
**Apgar 1 <7**	9 (14.8)	16 (7.7)	5 (9.3)	35 (16.1)	0	19 (9.0)	6 (7.3)	26 (6.5)	7 (17.1)	35 (14.8)	12 (30.0)	49 (13.6)
**Arterial umbilical cord pH <7.15**	9 (15.5)	15 (7.2)	10 (19.6)	35 (15.9)	1 (4.4)	15 (7.1)	10 (15.4)	47 (11.8)	6 (15.8)	37 (15.6)	4 (11.8)	51 (14.1)

* Ventouse.

## DISCUSSION

The potential benefits and risks of applying the GebStart-tool in primiparous women were investigated in these comparative analyses using data from participants of the GebStart-study and baseline data from all study centers. The findings indicated that study participants for whom the GebStart-tool was applied during their initial contact with the maternity hospital had a significantly reduced likelihood of receiving labor augmentation, epidural analgesia, and cesarean section, compared to baseline data collected prior to the recruitment of the GebStart-study. Furthermore, the odds for opioid administration and spontaneous vaginal birth were higher in women with the applied GebStart-tool compared to baseline data. Most results were stable across the majority of study sites, indicating robustness of findings and increasing their credibility.

The GebStart-tool was developed with the aim of providing a decision aid for advising primiparous women about the best place to stay during early labor^20^. Early but also late hospital admission was found to be associated with negative labor and birth outcomes in previous research^9-11^. It was assumed that the distinction between women who are well at home and those with increased support needs, could improve outcomes. Since the GebStart-tool is designed precisely to enable this distinction, the investigation of the potential benefits of applying the tool was planned from the conceptualization of the study^22^. Even though the results of the current analyses are promising, the benefits and risks of applying the GebStart-tool need to be reinvestigated in larger samples.

One of the major challenges of early labor is the very different ways in which this labor phase is experienced^2,27^. This may also be the reason for the different care needs reported in previous studies^5,8^. The Cochrane Review of Kobayashi et al.^17^ investigated assessment and support during early labor to improve birth outcomes in primiparous and multiparous women. The authors did not find a reduction in cesarean section rates and only a minimal decrease in labor augmentation rates with oxytocin and administering an epidural analgesia. In contrast, the current study provides evidence that the application of the GebStart-tool could reduce cesarean section, labor augmentation, and epidural analgesia. Kobayashi et al.^17^ concluded that early labor care needs to be more individualized and women-centered. The application of the GebStart-tool seems to be able to address this requirement. Previous studies also showed that early admission to hospital during the early labor process, but also admission after more than 24 hours of experiencing contractions at home, were associated with increased cesarean section rates^9,10,28^. In the current analysis, neither the cervical dilatation at hospital admission nor the duration of contractions before could be compared. Nevertheless, the development of the GebStart-tool was intended to provide an instrument that could identify both parturients who are well at home and can stay there as long as possible, and those with increased needs for whom delayed professional support could be stressful and a source of negative experiences^3,12,14^. It seems that considering many different aspects relevant to care needs in the GebStart-tool is promising to reduce intrapartal interventions and improve birth outcomes.

The application of the GebStart-tool is also in line with a more holistic, salutogenic approach to early labor care, since it is not limited to contractions but covers various relevant components^14^. Women during early labor wish to be taken seriously with their different physical and emotional symptoms, but also their attitudes, support at home, and the distance to the hospital^2,8^. The preliminary version of the GebStart-tool comprised 32 items. However, staff at the study centers complained about its length and the time effort^29^. This was a reason to shorten the GebStart-tool considerably to 15 items. Nevertheless, all aspects were retained to ensure that individual advice can still be provided^20^. This reduced tool will be handier, but its usefulness and effectiveness still need to be investigated. Further research is therefore necessary.

### Strengths and limitations

Strengths of this study were that baseline data were collected from women giving birth prior to the start of the GebStart-study. Women also gave birth before the study was introduced in the study sites. Nevertheless, the centers already agreed to participate in the study, and staff might have become sensitized to the challenges of early labor care. This potentially reduced differences between baseline and study data, strengthening these comparative analyses.

A limitation of the study was that some participants who underwent a cesarean section during early labor had to be excluded from the current analysis for reasons of comparability, as they could not be identified in the baseline data. Data from a respectable number of parturients could be included, but the sample size calculation for the GebStart-study was mainly conducted for scale development. It was adequately large to investigate interventions and events with a prevalence higher than 5%, but the sample size might have been too small for the comparison of rare events such as low Apgar scores and umbilical cord pH. A further limitation was the restricted availability of variables in the baseline data due to the considerable effort involved in data collection at most study centers. For example, no additional perinatal data were collected to further investigate neonatal outcomes. Furthermore, only limited analyses of continuous variables could be conducted, and no multivariable analyses to adjust odds ratios were possible due to the use of aggregated baseline data. Moreover, the comparison of gravity >1 and opioid administration could only be performed using data from five study sites due to missing data from one site for each variable.

Additionally, participants in the GebStart-study may have been very interested in giving birth physiologically, potentially leading to a selection bias. However, they could also have been afraid of early labor, which could have motivated study participation, leading to an opposite selection bias. These factors could weaken the results of the study, but they could also strengthen them. Furthermore, in the baseline data, no sociodemographic data were available, and so it was not possible to evaluate the comparability of the samples. It should also be noted that, in several study centers, baseline data were collected by screening the hospitals’ birth registers by hand. This was necessary, as no filter could be set for primiparous women with spontaneous onset of labor for data extraction from the electronic databases. This could have led to errors in data collection. Nevertheless, the robustness of most results in the study centers increases their credibility.

## CONCLUSIONS

Applying the preliminary GebStart-tool showed very promising results in reducing intrapartal interventions and improving birth outcomes. The development process of the GebStart-tool was based on scientific evidence and resulted in a tool that addresses various aspects that play an important role during early labor. This seems to have the potential to advise women individually and increase woman-centered care. Women may have perceived that their individual needs were acknowledged and taken seriously. This could have increased confidence and led to a less complicated labor and birth process. In a future larger study, preferably in a hybrid implementation study, the effectiveness of applying the final version of the GebStarttool should be investigated.

## Supplementary Material



## Data Availability

The data supporting this research are available from the authors on reasonable request.
